# Association of systemic immune-inflammation index with insulin resistance and prediabetes: a cross-sectional study

**DOI:** 10.3389/fendo.2024.1377792

**Published:** 2024-06-05

**Authors:** Han Guo, Chuan Wan, Jingjing Zhu, Xiuxing Jiang, Shufa Li

**Affiliations:** ^1^ Department of Endocrinology and Metabolism, The Affiliated Hospital of Qingdao University, Qingdao, China; ^2^ Frontier Medical Training Brigade, Third Military Medical University (Army Medical University), Xinjiang, China

**Keywords:** systemic immune-inflammation index, insulin resistance, prediabetes, cross-sectional study, NHANES

## Abstract

**Background and Objective:**

Previous research suggested a relationship between the Systemic Immune-Inflammation Index (SII) and multiple adverse health conditions. However, the role of SII in prediabetes and insulin resistance (IR) remains poorly understood. Therefore, this study aims to explore the potential relationship between SII and prediabetes and IR, providing data support for effective diabetes prevention by reducing systemic inflammation.

**Methods:**

Linear regression models were used to assess the correlation between continuous SII and risk markers for type 2 diabetes (T2D). Subsequently, multivariate logistic regression models and subgroup analyses were employed to evaluate the association between SII tertiles and prediabetes and IR, controlling for various confounding factors. Finally, restricted cubic spline graphs were used to analyze the nonlinear relationship between SII and IR and prediabetes.

**Results:**

After controlling for multiple potential confounders, SII was positively correlated with fasting blood glucose (FBG) (β: 0.100; 95% CI: 0.040 to 0.160), fasting serum insulin (FSI) (β: 1.042; 95% CI: 0.200 to 1.885), and homeostasis model assessment of insulin resistance (HOMA-IR) (β: 0.273; 95% CI: 0.022 to 0.523). Compared to participants with lower SII, those in the highest tertile had increased odds of prediabetes (OR: 1.17; 95% CI: 1.02-1.34; p for trend < 0.05) and IR (OR: 1.35; 95% CI: 1.18 to 1.51; p for trend<0.001).

**Conclusions:**

Our study results demonstrate an elevated association between SII levels and both IR and prediabetes, indicating SII as a straightforward and cost-effective method identifying individuals with IR and prediabetes.

## Introduction

Type 2 diabetes (T2D) poses a significant global public health challenge, profoundly affecting human health and quality of life (1). Prediabetes and insulin resistance (IR) stand out as primary contributors to T2D development, characterized by aberrant glucose metabolism (2). IR is a pivotal factor in various metabolic disorders, signifying a state where insulin-responsive tissues exhibit reduced responsiveness to physiological insulin levels. This state results in hyperinsulinemia and elevated fasting blood glucose (FBG), diagnostic indicators of IR (3). Despite being the gold standard for assessing IR, the hyperinsulinemic-euglycemic clamp is a costly, intrusive, and time-consuming procedure that needs to be performed by skilled staff. Therefore, the homeostasis model assessment of insulin resistance (HOMA-IR) offers a more straightforward and useful option by measuring insulin and fasting blood glucose (4).

IR is not only a key pathogenic factor in T2D but is also associated with various pathological conditions such as cardiovascular diseases, certain types of cancer, infertility, polycystic ovary syndrome, non-alcoholic fatty liver disease, and metabolic syndrome (5). Given the substantial harm that IR and prediabetes inflicts on human health, the early detection and intervention of them are hot topics among scholars in the relevant field. Previous research indicates that systemic chronic inflammation plays a pivotal role in IR and prediabetes, with obesity frequently triggering this inflammatory state (6). Obesity can induce a chronic inflammatory state in various insulin-target tissues, including adipose tissue, liver, muscles, and the pancreas. This is often attributed to potential interactions between immune processes and metabolic defects (7). Metabolic tissues induce the occurrence of this chronic low-grade inflammation by recruiting, accumulating, and activating pro-inflammatory macrophages. Although macrophages play a central role, other immune cell types are involved in these inflammatory processes (8). Satoshi discovered that CD8+ T cells can activate macrophages within adipose tissue. This alteration of the immune microenvironment leads to the shift of adipose tissue from an anti-inflammatory state to a pro-inflammatory state (9). Hence, controlling inflammation seems to be a pivotal intervention for mitigating IR and prediabetes. Nevertheless, studies on the population-level association between inflammation and IR and prediabetes are still limited. A comprehensive exploration of this potential connection necessitates an urgently required objective assessment indicator that precisely mirrors the immune and inflammatory status of populations with IR and prediabetes.

The systemic immune-inflammation index (SII), developed by Hu et al., is a novel, comprehensive biomarker for immune inflammation based on blood cells (10). It integrates three types of inflammatory cells-platelets, neutrophils, and lymphocytes. It accurately reflects the local immune response and systemic inflammatory status of body (11). Multiple studies have substantiated the prognostic value of SII in assessing outcomes for various cancer patients, encompassing bladder cancer (12), cervical cancer (11), non-small cell lung cancer (13), colorectal cancer (14), and gastric cancer (15). In recent years, SII has emerged as an indicator for detecting chronic inflammatory disease beyond tumors. Yang et al. reported that in patients with coronary artery disease after coronary intervention, SII demonstrates superior predictive value for major cardiovascular events compared to traditional risk factors (16). Wang et al. suggested using SII as an indicator for detecting diabetes depression (17). Additionally, several studies utilizing the NHANES database reveal a robust association between SII and various metabolic diseases, such as diabetic nephropathy (18), hepatic steatosis (19), and osteoporosis (20). Moreover, numerous studies indicate that, compared to traditional immune-inflammatory indicators [including lymphocyte/monocyte ratio (LMR), platelet/lymphocyte ratio (NLR)], SII is a more accurate predictor of malignant tumors (21, 22).

In summary, SII is a non-invasive quantitative indicator with higher research value compared to traditional inflammatory markers. Given the relationship between inflammation and IR as well as prediabetes, we hypothesize that a high SII level may be positively associated with the risk of developing IR and prediabetes. However, research in this field is currently limited. Therefore, this study plans to employ the NHANES database for a more rigorous statistical analysis methods, controlling confounding variables to validate this hypothesis. Our goal is to identify individuals at high risk of IR through SII and explore the association between SII and markers of T2D risk. This will help investigate its potential to identify prediabetic patients.

## Materials and methods

### Data source and study sample

The National Health and Nutrition Examination Survey (NHANES), conducted by the National Center for Health Statistics (NCHS), is a nationwide survey assessing the health and nutritional status of adults and children in the United States. It employs a cross-sectional, multi-stage, stratified, and sub-group probability sampling design, with a two-year cycle (23). The survey covers various aspects, including in-home face-to-face interviews (demographics, socioeconomic status, diet, and health-related questions), as well as health examinations conducted at Mobile Examination Centers (MEC) collecting medical data, anthropometry, and laboratory tests (24). The NHANES protocol is revised and approved by the NCHS Ethics Review Committee, and all participants provide written informed consent (25).

**Figure 1:**
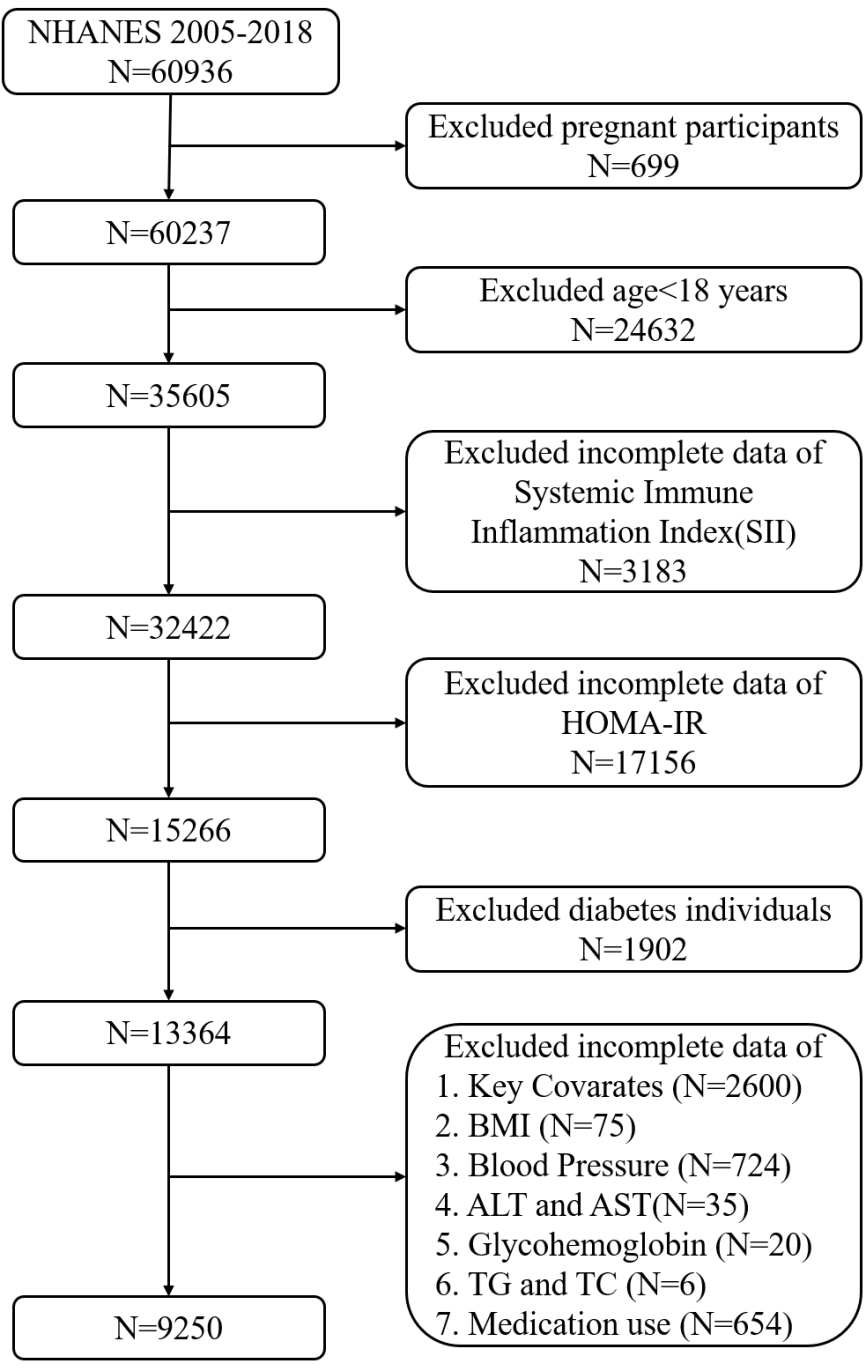
Flow chart of participants selection from the NHANES 2005-2018.

The population data used in this cross-sectional study are from the NHANES database, covering seven consecutive periods (2005-2006, 2007-2008, 2009-2010, 2011-2012, 2013-2014, 2015-2016, and 2017-2018). It involves 60,936 participants, consistent with the results of Liu, et al. (26). We excluded pregnant participants, those under 18, potential type 1 diabetes patients (defined as those <20 years receiving only insulin treatment), and T2D patients (self-reported diabetes, insulin or oral hypoglycemic medication use, HbA1c (≥6.5%), fasting blood glucose (≥126 mg/dL), or impaired glucose tolerance (≥200 mg/dL) (27), along with patients using various medications that may affect insulin sensitivity or with missing data (independent, dependent, and covariate data) (28). Finally, the study included 9,250 participants with complete data (**Figure 1**).

### Exposure variable and outcome variables

The exposure variable is SII, calculated as platelet count × neutrophil count/lymphocyte count (29). Subsequently, participants were divided into three groups based on the tertiles of SII, namely Tertile 1 (1.53≤SII<356.6), Tertile 2 (356.67≤SII<552.75), and Tertile 3 (SII≥552.75). Outcome variables include risk markers for IR, prediabetes, and T2D, such as fasting blood glucose (FBG), glycated hemoglobin (HbA1c), fasting serum insulin (FSI), and Homeostatic Model Assessment for Insulin Resistance (HOMA-IR). The formula for calculating HOMA-IR is [FBG × FSI/22.5] (30). In this study, HOMA-IR>2.6 is considered the diagnostic criterion for IR (31). Prediabetes is defined based on questionnaire and laboratory tests, with HbA1c levels between 5.7% and 6.4% or impaired fasting glucose levels (100-125 mg/dL) and/or impaired glucose tolerance (140-199 mg/dL) (32).

### Covariates

Provided participants with standardized questionnaires to collect sociodemographic and lifestyle information. Based on previous research, we included covariates related to metabolic health risk factors, including low socioeconomic status, smoking status, alcohol consumption, physical activity, systolic blood pressure (SBP), diastolic blood pressure (DBP), body mass index (BMI), total cholesterol (TC), serum triglycerides (TG), alanine aminotransferase (ALT), aspartate aminotransferase (AST), gamma-glutamyltransferase (γ-GGT), alkaline phosphatase (ALP), serum creatinine (Cr), and lactate dehydrogenase (LDH). This study also included gender (male or female), age as a continuous variable or categorical variable (18-39 years, 40-59 years, or ≥60 years), race/ethnicity (non-Hispanic Black, other Hispanic, non-Hispanic White, or other race), education level (less than high school, high school, and beyond high school), PIR (categorized as 1, 1-2, 2-4, and >4), smoking status categorized as never smoked (before the survey<100 cigarettes), former smoker (smoked >100 cigarettes before the survey but quit before the survey), and current smoker (smoked >100 cigarettes before the survey and smoked during the survey) (33). Participants who consumed at least 12 drinks of any type of alcoholic beverage (12 ounces of beer, 5 ounces of wine, or 1.5 ounces of distilled spirits) within the past year were classified as drinkers (34). MVPA was defined as completing at least 10 minutes of vigorous/moderate-intensity physical activity in a typical week (2007-2018 cycle), resulting in substantial sweating, or a significant increase in breathing or heart rate (35).

### Statistical analysis

In the description of the study population, continuous variables, if normally distributed, are presented as the mean with standard deviation; if skewed, they are presented as the median (25th-75th percentile). Both the normally and skewed distributed variables are analyzed using weight linear regression. Categorical variables are expressed as percentages and analyzed using the weighted chi-square test. We first established three weighted multivariable linear regression models to analyze the correlation between SII and T2D risk markers (FBG, HbA1c, FSI, and HOMA-IR). Model 1 was unadjusted for any covariates, Model 2 adjusted for covariates including age, gender, race, smoking and drinking status, PIR, education level, and physical activity status, and Model 3 adjusted for age, gender, race, smoking and drinking status, PIR, education level, physical activity status, BMI, TC, TG, ALT, AST, γ-GGT, ALP, Cr, and LDH. Subsequently, to evaluate the association between continuous LgSII and participants stratified into three tertiles and IR and prediabetes, we conducted a multifactorial logistic regression. Afterward, subgroup analyses were performed to test for interaction and control of confounding categorical variables, including age (18-39 years, 40-59 years, or ≥60 years), gender, race, education level, PIR, smoking status, alcohol consumption, and MVPA. Subgroup analyses used weighted multifactorial logistic regression. These stratification variables were also considered predefined effect-modifying factors. To examine the heterogeneity of associations between subgroups, interaction terms were introduced. Finally, we used Restricted Cubic Spline (RCS) regression to examine the non-linear relationship between SII and IR and prediabetes. Likelihood ratio tests were employed to confirm this relationship. It is noteworthy that, during regression analysis, SII was log-transformed as it exhibited a right-skewed distribution. All analyses were conducted using R software (version 4.1.2).

## Results

### Baseline characteristics of the study population

**Table 1** presents the baseline characteristics of participants categorized by SII status. The study comprised 9250 participants, including 4827 males and 4423 females, with a median age of 45 years. Among them, 43.11% were diagnosed with IR, and 19.7% were considered to have prediabetes. Participants were divided into groups based on SII tertiles: Tertile 1 represented the relatively lower SII group (1.53≤SII<356.67); Tertile 2 represented the relatively higher SII group (356.67≤SII<552.75); Tertile 3 represented the highest SII group (SII≥552.75). Compared to those in the lower SII group, subjects in SII Tertile 3 included more females, fewer non-Hispanic Blacks, more current or former smokers, individuals with lower educational attainment, and those engaged in less physical activity. Across the three SII groups, significant differences were observed in BMI, FBG, HbA1c, FSI, HOMA-IR, TC, TG, ALT, AST, chloride, SBP, alkaline phosphatase, and creatinine levels (all P values <0.05). Importantly, individuals with higher SII levels had a higher proportion of prediabetes and IR patients (P<0.05 in both cases).

**Table 1:** Basic characteristics of the study population (n=9250) in the NHANES.

Variables	Overall	Tertile 1	Tertile 2	Tertile 3	P value
Categorical variables l% (No.)					
Gender					<0.001
Male	52.18 (4827)	56.24 (1874)	50.94 (1686)	45.79 (1521)	
Female	47.82 (4423)	43.76 (1450)	49.06 (1624)	54.21 (1801)	
Race/Ethnicity					<0.001
Mexican American	15.32 (1417)	14.77 (492)	16.71 (553)	14.09 (468)	
Other Hispanic	9.33 (863)	8.34 (278)	9.85 (326)	9.30 (309)	
Non-Hispanic White	47.48 (4392)	39.41 (1313)	48.79 (1615)	55.75 (1852)	
Non-Hispanic Black	18.36 (1698)	26.05 (868)	15.29 (506)	13.46 (447)	
Other Races	9.51 (880)	11.43 (381)	9.37 (310)	7.41 (246)	
Education levels					0.003
Less than high school	22.96 (2124)	22.69 (756)	23.26 (770)	24.02 (798)	
High school diploma	23.29 (2154)	23.50 (783)	21.33 (706)	25.05 (832)	
More than high school	53.75 (4972)	53.81 (1973)	55.41 (1834)	50.93 (1692)	
PIR					0.032
<1	20.56 (1902)	21.01 (700)	19.58 (648)	21.70 (721)	
1-2	25.22 (2333)	25.39 (846)	24.92 (825)	26.31 (874)	
2-4	27.26 (2522)	26.98 (899)	26.92 (891)	27.36 (909)	
>4	26.95 (2493)	26.62 (887)	28.58 (946)	24.62 (818)	
MVPA	76.94 (7117)	78.69 (2622)	78.43 (2596)	71.88 (2388)	<0.001
Alcohol consumption	73.99 (6844)	72.42 (2413)	73.93 (2447)	73.90 (2455)	0.279
Smoking status					<0.001
Current smoker	21.69 (2006)	19.00 (633)	19.61 (649)	26.34 (875)	
Non-smoker	55.10 (5097)	57.86 (1928)	56.62 (1874)	49.16 (1633)	
Former smoker	23.21 (2147)	23.14 (771)	23.78 (787)	24.50 (814)	
Insulin resistance					<0.001
Yes	43.11 (3988)	40.15 (1335)	43.22 (1427)	48.07 (1595)	
No	56.89 (5262)	59.85 (1990)	56.78 (1875)	51.93 (1723)	
Continuous [Mean ± SD, Median (IQR)]					
Age (years)	45.00 (32.00-61.00)	44.00 (30.00-60.00)	44.00 (32.00-60.00)	47.00 (34.00-63.00)	<0.001
BMI (kg/m^2^)	28.38 ( ± 6.56)	27.58 ( ± 5.91)	28.45 ( ± 6.26)	29.14 ( ± 7.35)	<0.001
ALT (U/L)	21.00 (16.00-28.00)	21.00 (17.00-29.00)	21.00 (16.00-29.00)	20.00 (16.00-27.00)	<0.001
AST (U/L)	20.00 (23.00-28.00)	24.00 (20.00-28.00)	23.00 (20.00-28.00)	22.00 (19.00-27.00)	<0.001
Glycohemoglobin (%)	5.45 ( ± 0.47)	5.45 ( ± 0.47)	5.43 ( ± 0.45)	5.48 ( ± 0.48)	0.009
TC (mmol/L)	5.03 ( ± 1.05)	4.96 ( ± 1.05)	5.08 ( ± 1.05)	5.05 ( ± 1.04)	<0.001
TG (mmol/L)	1.12 (0.77-1.66)	1.03 (0.72-1.59)	1.14 (0.79-1.69)	1.16 (0.81-1.69)	<0.001
SBP (mmHg)	122.57 ( ± 17.84)	122.14 ( ± 17.83)	121.97 ( ± 17.50)	123.63 ( ± 18.13)	0.001
DBP (mmHg)	69.39 ( ± 12.77)	69.35 ( ± 12.19)	69.54 ( ± 12.51)	69.27 ( ± 13.59)	0.810
Gamma glutamyl transferase (U/L)	19.00 (14.00-29.00)	19.00 (14.00-29.00)	19.00 (14.00-29.00)	19.00 (14.00-30.00)	0.152
Alkaline phosphotase (U/L)	68.02 ( ± 22.57)	65.70 ( ± 22.95)	67.36 ( ± 21.12)	71.04 ( ± 23.27)	<0.001
Lactate dehydrogenase (U/L)	128.62 ( ± 32.05)	128.83 ( ± 37.81)	127.59 ( ± 26.45)	129.45 ( ± 30.70)	0.462
Chloride (mmol/L)	104.13 ( ± 2.73)	104.20 ( ± 2.61)	104.19 ( ± 2.71)	103.99 ( ± 2.87)	0.003
Creatinine (μmol/L)	75.14 (63.65-88.40)	76.91 (65.20-88.40)	73.37 (62.76-87.52)	73.37 (61.88-88.40)	0.005
Fasting Glucose (mmol/L)	5.57 ( ± 0.76)	5.52 ( ± 0.73)	5.57 ( ± 0.76)	5.62 ( ± 0.78)	<0.001
Insulin (μU/mL)	9.31 (5.98-14.96)	8.77 (5.64-14.15)	9.27 (6.00-14.92)	10.02 (6.34-16.05)	<0.001
HOMA-IR	2.13 (1.34-3.55)	2.27 (1.41-3.85)	2.49 (1.49-4.12)	2.51 (1.49-4.19)	<0.001

Mean ± SD for continuous variables. The percentage (95% CI) for categorical variables.

SII, Systemic Immune-Inflammation Index; PIR, poverty income ratio; BMI, body mass index; MVPA, moderate/vigorous physical activity; SBP, systolic blood pressure; DBP, diastolic blood pressure; ALT, alanine aminotransferase; AST, aspartate aminotransferase; TC, total cholesterol; TG, triglycerides.

### Association between SII and T2D risk markers

The correlation between SII and the risk markers of T2D was showed in **Table 2**. After adjusting for potential confounders, a significant correlation was observed between continuous SII and FBG, FSI, and HOMA-IR, while the relationship between SII and HbA1c was significant only when no covariates were adjusted. Following comprehensive adjustment for covariates in Model 3, participants in the second or third tertiles of SII had higher levels of FBG, FSI, and HOMA-IR compared to those in the first tertile. And the corresponding β coefficients (95% CI) for the highest tertile of SII were 0.048 (95% CI: 0.014 to 0.082), 0.658 (95% CI: 0.175 to 1.141), and 0.165 (95% CI: 0.021 to 0.309) for FBG, FSI, and HOMA-IR, respectively.

**Table 2:** Coefficients (95% CI) for the relationship between the SII and the markers of T2D risk.

Outcomes	β (95% CI) Per SD increase	Tertile 1	Tertile 2	Tertile 3	P for trend
FBG					
Model 1	0.185 (0.120,0.251)	Reference	0.045 (0.008,0.083)	0.099 (0.061,0.137)	<0.001
Model 2	0.149 (0.087,0.211)	Reference	0.047 (0.012,0.083)	0.077 (0.041,0.113)	<0.001
Model 3	0.100 (0.040,0.160)	Reference	-0.025 (-0.008,0.059)	0.048 (0.014,0.082)	0.007
HOMAIR					
Model 1	0.580 (0.303,0.858)	Reference	0.120 (-0.039,0.279)	0.358 (0.198,0.518)	<0.001
Model 2	0.553 (0.274,0.831)	Reference	0.119 (-0.039,0.277)	0.342 (0.182,0.502)	<0.001
Model 3	0.273 (0.022,0.523)	Reference	-0.024 (-0.165,0.117)	0.165 (0.021,0.309)	0.014
FSI					
Model 1	2.047 (1.107,2.988)	Reference	0.382 (-0.156,0.920)	1.290 (0.750,1.831)	<0.001
Model 2	2.035 (1.093,2.977)	Reference	0.378 (-0.157,0.912)	1.288 (0.747,1.829)	<0.001
Model 3	1.042 (0.200,1.885)	Reference	-0.124 (-0.598,0.350)	0.658 (0.175,1.141)	0.003
HAb1c					
Model 1	0.043 (0.003,0.084)	Reference	-0.015 (-0.039,0.008)	0.031 (0.008,0.055)	0.003
Model 2	-0.0002 (-0.038,0.037)	Reference	-0.014 (-0.035,0.007)	0.002 (-0.019,0.024)	0.662
Model 3	-0.043 (-0.079,-0.006)	Reference	-0.028 (-0.049,-0.008)	-0.019 (-0.041,0.002)	0.132

### Relationship between SII and IR

As shown in **Table 3**, a significant positive correlation between SII and IR was demonstrated in the unadjusted original model (Model 1) (odds ratio=1.73; 95% confidence interval (CI), 1.45-2.06; P<0.001) and in the minimally adjusted model (Model 2) (OR=1.89; 95% CI: 1.57-2.28; P<0.001). Even after adjusting for all covariates (Model 3), this positive correlation persisted (OR=1.64; 95% CI: 1.32-2.04; P<0.001). This implies that for every one-unit increase in LgSII, the risk of IR increases by 64%. We further transformed SII from a continuous variable to a categorical variable (tertiles) for sensitivity analysis. Compared to participants in the lowest Tertile 1 group of SII, those in the highest Tertile 3 group had a 34% increased risk of IR, with statistical significance (OR=1.34; 95% CI: 1.18-1.51; P<0.001). Although participants in the Tertile 2 group also exhibited a higher risk of IR compared to the Tertile 1 group, with a 4% increased risk, but this difference was not statistically significant.

**Table 3:** The associations between SII and IR.

	Model 1		Model 2		Model 3	
	OR (95% CI)	P value	OR (95% CI)	P value	OR (95% CI)	P value
Continuous LgSII	1.73 (1.45, 2.06)	<0.001	1.89 (1.57, 2.28)	<0.001	1.65 (1.32, 2.04)	<0.001
Tertile 1	Reference		Reference		Reference	
Tertile 2	1.14 (1.03, 1.26)	0.011	1.18 (1.06, 1.31)	0.002	1.04 (0.92, 1.18)	0.510
Tertile 3	1.40 (1.27, 1.55)	<0.001	1.47 (1.32, 1.63)	<0.001	1.34 (1.18, 1.51)	<0.001
P for trend	<0.001		<0.001		<0.001	

### Relationship between SII and prediabetes

**Table 4** presents the association between SII evaluated based on its tertiles and prediabetes. In Model 1, without adjusting for any factors, the relationship between continuous LgSII and prediabetes was significant (OR=1.60; 95% CI: 1.29-2.00; P<0.001). After adjusting for all potential covariates, an increase of 1 unit in SII score was associated with a 43% increased odds of having prediabetes (OR: 1.43; 95% CI: 1.13 to 1.82). Additionally, we assessed the relationship between tertiles of SII scores and prediabetes. Individuals with the highest SII tertile had a 17% increased odds of prediabetes compared to those in the lowest tertile (OR: 1.17; 95% CI: 1.02 to 1.34; P=0.029), whereas the association between the second SII tertile and prediabetes was not significant.

**Table 4:** The associations between SII and prediabetes.

	Model 1		Model 2		Model 3	
	OR (95% CI)	P value	OR (95% CI)	P value	OR (95% CI)	P value
Continuous LgSII	1.60 (1.29, 2.00)	<0.001	1.61(1.28,2.03)	<0.001	1.43 (1.13,1.82)	0.003
Tertile 1	Reference		Reference		Reference	
Tertile 2	1.01 (0.89, 1.15)	0.846	1.08 (0.95,1.24)	0.252	1.01 (0.88, 1.16)	0.865
Tertile 3	1.26 (1.12, 1.43)	<0.001	1.26 (1.10,1.44)	<0.001	1.17 (1.02, 1.34)	0.029
P for trend	<0.001		0.001		0.019	

### The multivariable logistic regression model

In the fully adjusted multivariable logistic regression model, age, race, smoking, alcohol consumption, physical activity, ALT, AST, alkaline phosphatase, BMI, triglycerides, and blood chloride levels still show significant associations with the odds of IR (**Table 5**). Factors significantly associated with the risk of prediabetes include age, gender, race, ALT, AST, BMI, TG, blood chloride levels, and LDH (**Table 6**). Compared to participants aged 18-39, those aged 40-59 have 2.45 times higher odds of prediabetes, while participants over 60 have 7.04 times higher odds of prediabetes (P<0.001), and a 35.5% higher risk of IR (P<0.001). Compared to Mexican-American individuals, non-Hispanic white individuals have a 27.8% lower likelihood of IR and a 28.2% lower likelihood of prediabetes (P<0.001), while non-Hispanic black individuals have a 24.3% higher risk of IR (P=0.02). Compared to smokers, non-smokers and former smokers have a 50.9% and 56.5% increased odds of IR, respectively (P<0.001).With each unit increase in TG and ALT, the odds of IR increase by 292.2% (P<0.001) and 26% (P<0.001), respectively, while the odds of prediabetes increase by 53.6% and 1% (both P<0.001). Compared to non-obese participants with a BMI less than 30, obese participants have 448% higher odds of IR and 99.8% higher odds of prediabetes (P<0.001).

**Table 5:** Multivariate logistic regression models of IR.

Variables	OR (95% CI)	P value
SII	1.645 (1.324, 2.042)	<0.001
Age (versus 18-39 years old)		
40-59	1.074 (0.954, 1.208)	0.237
>60	1.355 (1.162, 1.580)	<0.001
Female (versus male)	0.904 (0.805, 1.015)	0.087
Race (versus Mexican American)		
Other Hispanic	0.957 (0.782, 1.170)	0.668
Non-Hispanic White	0.722 (0.618, 0.845)	<0.001
Non-Hispanic Black	1.239 (1.033, 1.487)	0.021
Other Races	0.901 (0.730, 1.112)	0.332
Education level (versus less than high school)		
High school diploma	0.965 (0.832, 1.118)	0.635
More than high school	0.963 (0.837, 1.107)	0.593
Smoke (versus current smoker)		
Non-smoker	1.509 (1.316, 1.732)	<0.001
Former smoker	1.565 (1.340, 1.828)	<0.001
MVPA (versus less physical activity)	1.269 (1.127, 1.428)	<0.001
Alcohol consumption (versus less than 12 drinks/year)	1.202 (1.066, 1.356)	0.003
PIR (versus less than 1)		
1-2	0.945 (0.816, 1.094)	0.447
2-4	0.960 (0.826, 1.116)	0.597
>4	0.892 (0.759, 1.049)	0.167
Alkaline phosphotase (U/L)	1.004 (1.002, 1.006)	<0.001
ALT (U/L)	1.026 (1.021, 1.031)	<0.001
AST (U/L)	0.986 (0.979, 0.992)	<0.001
BMI (kg/m^2^)		
≥30	5.482 (4.922, 6.107)	<0.001
TC (mmol/L)	0.797 (0.756, 0.840)	<0.001
TG (mmol/L)	3.922 (3.519, 4.370)	<0.001
Chloride (mmol/L)	1.024 (1.004, 1.043)	0.012
Creatinine (mol/L)	0.998 (0.996, 1.000)	0.049
Gamma glutamyl transferase (U/L)	1.000 (0.999, 1.002)	0.664
Lactate dehydrogenase (U/L)	0.998 (0.996, 1.000)	0.067

**Table 6:** Multivariate logistic regression models of prediabetes.

Variables	OR (95% CI)	P value
SII	1.442 (1.136, 1.830)	0.003
Age (versus 18-39 years old)		
40-59	3.451 (2.974, 4.006)	<0.001
>60	7.041 (5.894, 8.411)	<0.001
Female (versus male)	0.625 (0.548, 0.711)	<0.001
Race (versus Mexican American)		
Other Hispanic	0.880 (0.702, 1.104)	0.269
Non-Hispanic White	0.718 (0.603, 0.886)	<0.001
Non-Hispanic Black	1.233 (1.007, 1.509)	0.042
Other Races	0.907 (0.706, 1.165)	0.444
Education level (versus less than high school)		
High school diploma	0.992 (0.845, 1.164)	0.918
More than high school	0.847 (0.726, 0.988)	0.035
Smoke (versus current smoker)		
Non-smoker	0.890 (0.762, 1.038)	0.138
Former smoker	0.935 (0.790, 1.106)	0.431
MVPA (versus less physical activity)	1.024 (0.901, 1.164)	0.714
Alcohol consumption (versus less than 12drinks/year)	1.108 (0.967, 1.268)	0.140
PIR (versus less than 1)		
1-2	0.934 (0.793, 1.101)	0.419
2-4	0.937 (0.790, 1.113)	0.460
>4	0.900 (0.749, 1.083)	0.266
Alkaline phosphotase (U/L)	1.001 (0.999, 1.004)	0.360
ALT (U/L)	1.010 (1.005, 1.075)	<0.001
AST (U/L)	0.986 (0.980, 0.993)	<0.001
BMI (kg/m^2^)		
≥30	1.988 (1.766, 2.237)	<0.001
TC (mmol/L)	0.974 (0.921, 1.031)	0.368
TG (mmol/L)	1.536 (1.375, 1.715)	<0.001
Chloride (mmol/L)	0.963 (0.944, 0.982)	<0.001
Creatinine (μmol/L)	0.998 (0.996, 1.000)	0.061
Gamma glutamyl transferase (U/L)	1.001 (0.999, 1.002)	0.278
Lactate dehydrogenase (U/L)	1.004 (1.002, 1.006)	<0.001

### Subgroup analysis

**Figure 2:**
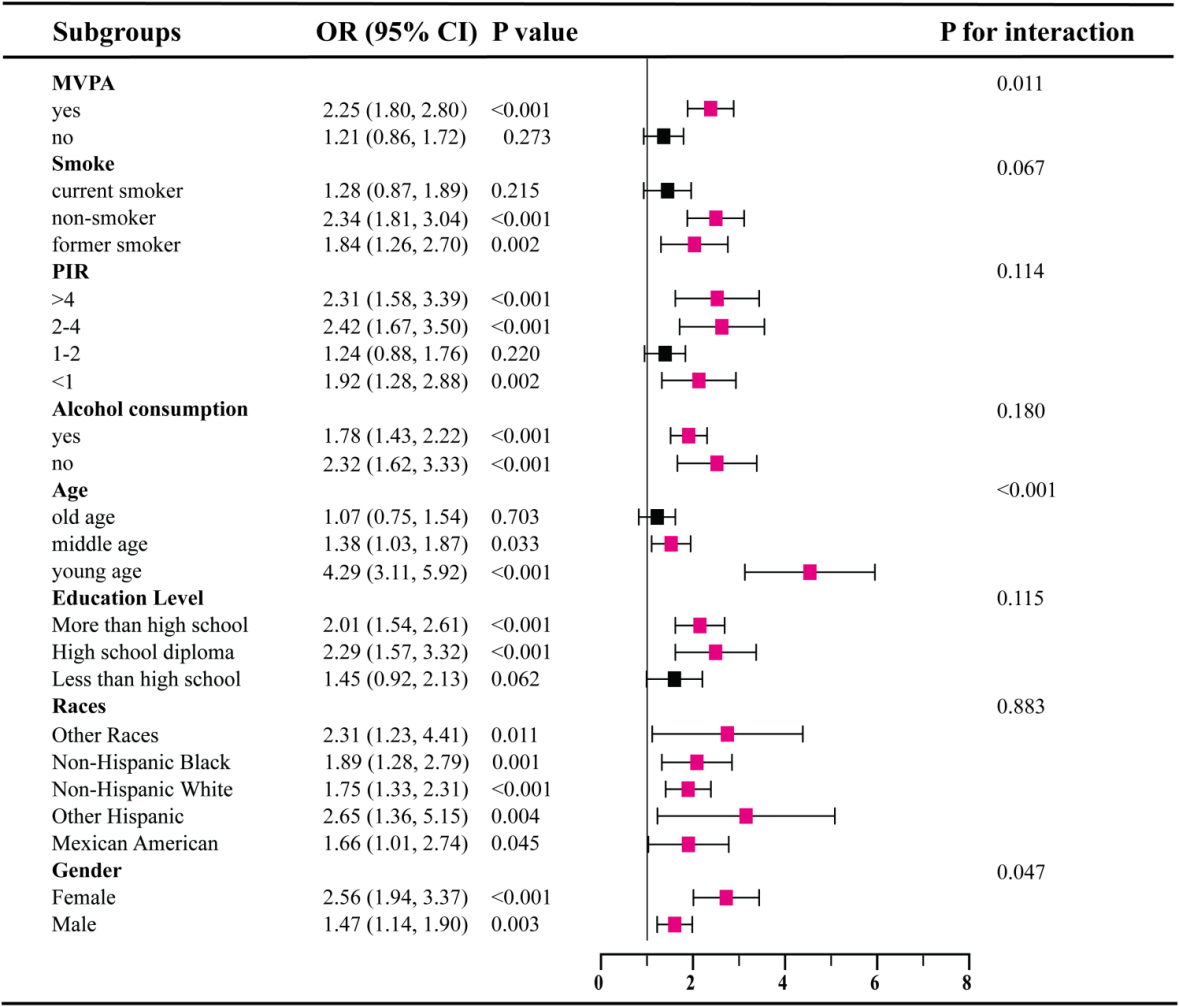
Subgroup analysis for the association between SII and IR.

**Figure 3:**
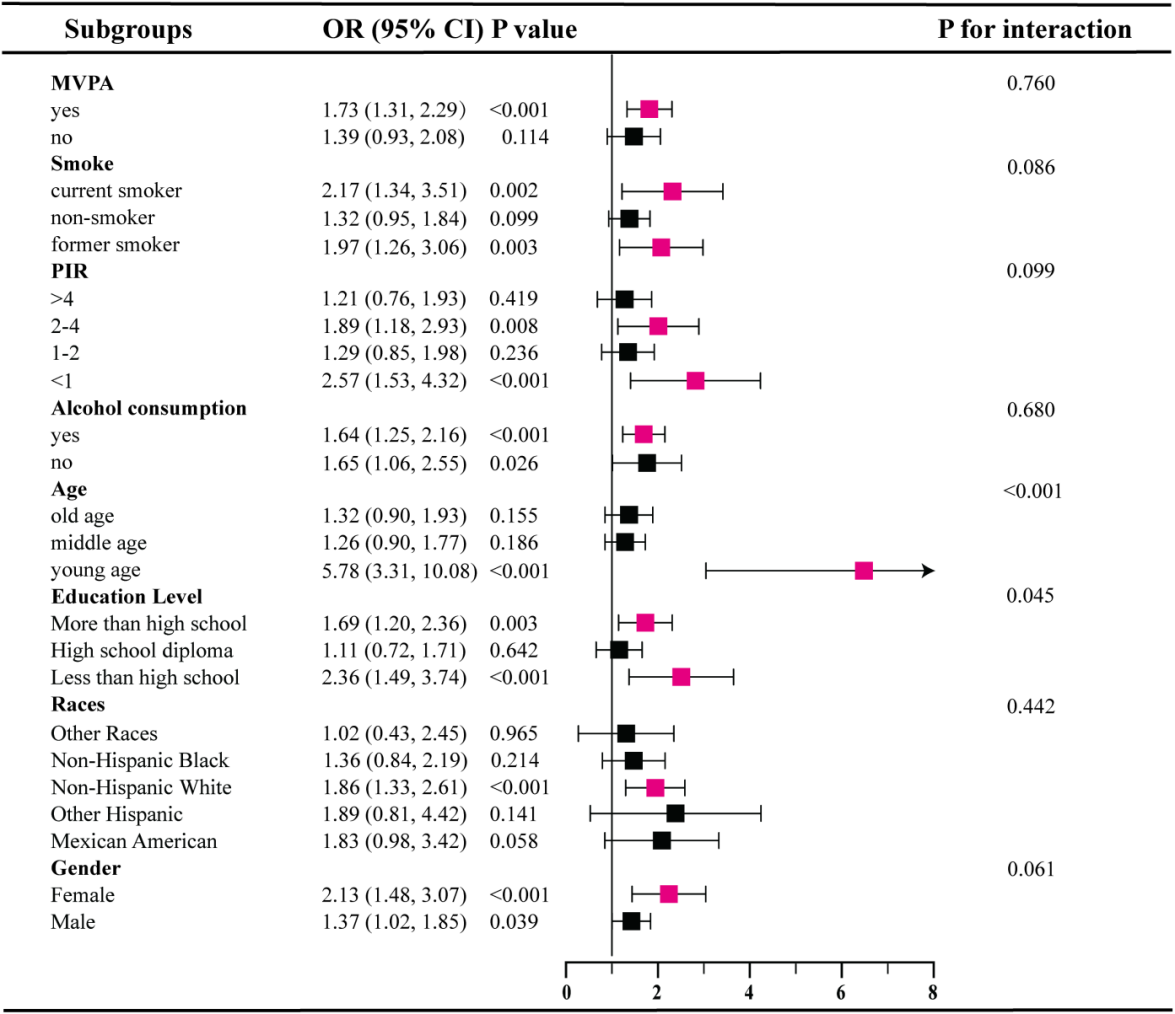
Subgroup analysis for the association between SII and Prediabetes.

Our subgroup analysis reveals inconsistent associations between SII levels and IR as well as prediabetes (**Figures 2**, **3**). In subgroups based on gender, race, and alcohol consumption, significant associations between SII and IR are detected in each subgroup (all P<0.05).However, among participants with prediabetes, only females, those aged 18-39, non-Hispanic white individuals, those with high school education or less, PIR<1 and 2<PIR<4, former or current smokers, drinkers, and physically active participants show statistically significant associations in subgroups stratified by gender, age, race, education, PIR, smoking status, alcohol consumption, and physical activity. Additionally, interaction tests show that age is the most prominent interacting factor influencing the relationship between SII and IR as well as prediabetes. For younger participants (18-39 years old), with increasing SII levels, the risk of both IR (OR=4.29, 95% CI: 3.11-5.92) and prediabetes (OR=5.78, 95% CI: 3.31-10.08) is significantly higher than in middle-aged participants (P<0.001).Among other factors, physical activity and gender are prominent factors influencing the relationship between SII and IR (P<0.05), and education level may influence the positive correlation between SII and prediabetes (P=0.045).

### Analysis of restricted cubic spline regression

**Figure 4:**
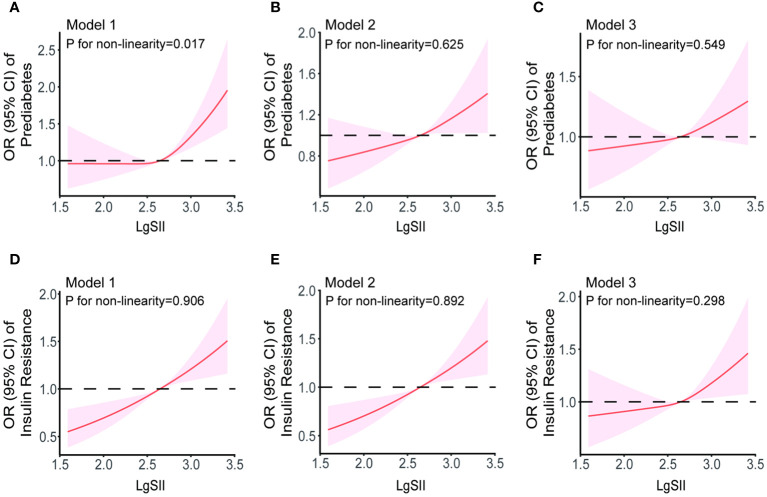
The dose-response relationships of SII with prediabetes **(A–C)** and IR **(D–F)** in Model 1 (unadjusted for any covariates), Model 2 (adjusted for covariates including age, gender, race, smoking and drinking status, PIR, education level, and physical activity status) and Model 3 (adjusted for age, gender, race, smoking and drinking status, PIR, education level, physical activity status, BMI, TC, TG, ALT, AST, γ-GGT, ALP, Cr, and LDH). Results were from restricted cubic spline models.

We further assessed the dose-response relationship between SII and prediabetes as well as IR using restricted cubic splines. In a model without adjusting for any covariates, we found a significant non-linear relationship between SII and prediabetes (P=0.017, **Figure 4A**), and the dose-response curve exhibits an inverted U shape. However, after adjusting for several covariates, the relationship between SII and prediabetes became linear (P>0.05) (**Figures 4B, C**). Additionally, irrespective of covariate adjustments, there is a linear dose-response relationship between SII scores and IR (**Figures 4D–F**).

## Discussion

To our knowledge, this is the initial investigation assessing the association between SII and prediabetes in the adults of the United States. A recent study have reported a positive linear associations between higher SII and increased risk of IR, which is consistent with our findings. However, their study did not adequately exclude patients with existing diabetes or those taking medications that could affect IR, and our research included a higher number of study populations (23). Therefore, our research results are more reliable and more complete.

In this cross-sectional study involving American adults, we observed a significant correlation between elevated SII levels and risk markers for T2D (FBG, FSI, and HOMA-IR). Furthermore, after further adjusting for potential confounding factors, the association between SII and increased risk of IR and prediabetes persisted. However, the positive correlation between SII and IR or prediabetes is more significant in females than in males. Additionally, the restricted cubic spline model indicates a linear dose-response relationship between SII and the odds of IR and prediabetes. These findings suggest that SII may serve as a monitoring indicator for IR and prediabetes.

Zhao et al. illustrated that continuous SII exhibit a skewed distribution, aligning with the structure of our original data. To approximate it to a normal distribution, they recommended logarithmic transformation of SII (24). From **Table 1**, it can be seen that some traditional diabetes risk factors, such as older age, smoking, and higher BMI, are more likely to have higher SII values, while protective factors like MVPA are more likely to have lower SII values. Additionally, our study revealed a positive correlation between continuous SII and glycated hemoglobin. However, this correlation vanished after adjusting for confounding factors and categorizing SII into tertiles.

SII is a recognized indicator for predicting cancer treatment outcomes and prognosis. Apart from cancer, the predictive value of SII for other metabolic-related diseases, such as diabetes and cardiovascular diseases, is also gaining attention (25, 26). The study by Nie et al. revealed an association between increased SII and increased prevalence of diabetes (27). Bian et al. found that CAD patients undergoing PCI with worse prognostic outcomes tended to have higher SII values (28). In a cross-sectional study conducted in the United States with 12,402 participants, a significant correlation between SII and metabolic syndrome was found after inclusion (24). Metabolic syndrome is a condition characterized by an aggregation of various metabolic risk factors related to IR and impaired glucose regulation (29). Inspired by this discovery, we focus on whether SII holds equal value in identifying and predicting IR and prediabetes. There are few studies that have independently evaluated the association between SII and IR as well as prediabetes. Numerous studies have indicated a significant association between SII and complications of diabetes.

In the work by Elbeyli, the SII was identified as a potential diagnostic biomarker for diabetic macular edema, with positive implications for improving diabetic retinopathy (30). Özata et al. further explored the correlation between SII and diabetic macular edema, revealing that an elevated SII level might lead to an increased incidence of serous retinal detachment (31). The research of Safak et al. unveiled the potential of SII as a predictive indicator for diabetic foot osteomyelitis (32). Moreover, in a survey of Indonesian diabetic patients, Yohanes and Andy found a significant association between low SII levels and the regulation of psychological well-being in diabetic patients (33).

In clinical studies, both IR and prediabetes have been found to be associated with several traditional inflammatory markers. Jia et al. used the rate nephelometry method to measure serum IMA and hs-CRP concentrations in patients with diabetic retinopathy, finding a positive correlation between hs-CRP concentration and the incidence of diabetic retinopathy (34). Liu et al. suggested that serum hs-CRP concentration can predict the incidence of diabetes (35). However, some studies have found no difference in the presence or absence of hs-CRP with IR. Systemic levels of TNF-a, IL-1b, IL-6, and CRP are elevated in both type 1 and type 2 diabetes patients (36, 37), which is a result of the chronic activation of pro-inflammatory pathways within insulin-target cells (38). Consequently, these cytokines and inflammatory mediators, especially TNF-α, monocyte chemoattractant protein-1 (MCP-1), CRP, and interleukins, are considered potential contributors to IR or impaired B-cell function (39). Additionally, in the study by Shu et al., it was found that the dietary inflammatory index is positively correlated with FBG, FSI, and HOMA-IR, and a more pro-inflammatory diet is associated with increased odds of IR and prediabetes (40).

In recent years, an increasing number of studies have focused on the significance of common inflammatory markers in blood routine examinations in the diagnosis and treatment of metabolic diseases. Additionally, Christine Lee and colleagues investigated the relationship between various white blood cell subtypes and IR in high-risk individuals, finding a positive correlation with all white blood cell subtypes, including granulocytes, lymphocytes, and monocytes (41). Karakaya studied 96 obese patients and 40 healthy controls, discovering a positive correlation between IR and white blood cell count, with NLR higher in obese IR patients than non-IR obese patients (42). Rodríguez-Rodríguez et al. also found a similar phenomenon in children (43). Some studies suggest that participants resistant to insulin have significantly higher platelet counts than insulin-sensitive participants (44). Hwang et al. found that with an increase in platelet count, the incidence of diabetes also increased, indicating that platelets are a potential risk marker (11). However, in the study by Rodríguez-Rodríguez, it was found that high platelet values do not constitute a risk factor for the occurrence of IR in children, and no relationship was observed between IR and PLR (43). In related reports, it seems that NLR is better at predicting inflammation than PLR, as neutrophils play a dominant role in inflammation by releasing vasoactive and cytotoxic substances (such as reactive oxygen species and digestive enzymes) during inflammation, leading to increased endothelial permeability (45). Therefore, it is necessary to introduce a more accurate SII index that includes platelet count for further research.

In various studies on inflammatory markers, SII, with its advantage of integrating three key immune cells, provides a more comprehensive description of the body’s inflammatory state compared to traditional single inflammatory biomarkers. For instance, Berbudi et al. revealed that SII, in predicting the impact of T2D on the immune system, demonstrates more precise and effective predictive capability than NLR, PLR, and MLR, as significantly confirmed by ROC curve analysis (46). Furthermore, the study by Nicoară’s team confirms that, in differentiating whether obese children have metabolic syndrome, SII exhibits higher diagnostic efficacy compared to NLR, PLR, and SIRI. It also shows a positive correlation with the HOMA-IR (47). These findings offer a new perspective for clinically assessing inflammatory and metabolic abnormal states.

The primary strength of this study is the inclusion of a large number of samples. Based on NHANES database, we analyzed a total of 9250 samples from 2005 to 2018, including self-reports, laboratory tests, and physical examinations. These data were collected by professionals through a standardized procedure, significantly reducing errors caused by different methods. The enormous sample size and standardized data contribute to obtaining meaningful and highly reliable results even after multiple condition screenings. And this study not only screened diabetic patients based on fasting blood glucose level >7.0 mmol/L but also excluded potential diabetic patients based on medication use and HbA1c levels. Additionally, to explore the impact of confounding factors on the association between SII and IR and prediabetes, we conducted stratified analyses. The results revealed that factors such as age, gender, race, education level, and family income-poverty ratio had some impact on the incidence of IR and prediabetes. For example, older patients have higher SII values and are at higher risk for IR and prediabetes. And our results show that women are more likely to have higher SII values than men, suggesting that they are more likely to have IR as well as pre-diabetes. Furthermore, lifestyle factors such as smoking, more alcohol consumption, and lower physical activity level were also positively associated with the risk of developing IR and prediabetes. Finally, we used non-restrictive cubic spline plots to analyze the nonlinear relationship between SII and IR and prediabetes, providing a more comprehensive understanding of their development and risk factors.

However, this study has certain limitations. Firstly, the nature of observational research restricts our analysis of causal relationships, leaving room for multiple interpretations, including both causation and reverse causation. Therefore, prospective studies are urgently needed to clarify the precise connections among these factors. Additionally, despite adjusting for various covariates, potential confounding factors such as dietary patterns and a family history of T2D might still be overlooked. It’s worth noting that, although diet has a significant impact on circulating TG levels, fasting blood samples collected in this study may not fully capture this aspect. Future research should delve into exploring the specific influence of dietary factors on study results.

Despite some limitations and drawbacks, our study still holds significant clinical relevance. As a novel inflammatory biomarker, SII not only offers the advantage of non-invasiveness but also provides a more comprehensive approach to assessing immune and inflammatory responses. The results of this study confirm our previous hypothesis that SII can serve as crucial indicators for diagnosing IR and prediabetes. In an era where IR is prevalent, often elusive, and troubling to primary care communities, the SII scoring system, comprising three simple and efficient hematological indicators, provides a practical diagnostic tool for primary healthcare practitioners. In the future, we plan to initiate a multicenter prospective cohort study to explore the effectiveness of SII as an independent predictor of IR and prediabetes. The goal is to offer prospective guidance and intervention for high-risk individuals through routine blood cell count monitoring, aiming for prevention and protection.

## Conclusion

In conclusion, this study indicates a positive correlation between SII and FBG, FSI, and HOMA-IR, so higher SII levels may increase the odds of IR and prediabetes. Therefore, SII is poised to be a direct and cost-effective method for identifying patients with IR and prediabetes.

## Data availability statement

The datasets presented in this study can be found in online repositories. The names of the repository/repositories and accession number(s) can be found below: https://www.cdc.gov/nchs/nhanes/.

## Ethics statement

The studies involving humans were approved by National Center for Health Statistics. The studies were conducted in accordance with the local legislation and institutional requirements. The human samples used in this study were acquired from gifted from another research group. Written informed consent for participation was not required from the participants or the participants’ legal guardians/next of kin in accordance with the national legislation and institutional requirements. Written informed consent was obtained from the individual(s) for the publication of any potentially identifiable images or data included in this article.

## Author contributions

HG: Writing – original draft, Validation, Methodology, Data curation. CW: Writing – original draft, Methodology. JZ: Writing – original draft. XJ: Writing – review & editing, Project administration. SL: Writing – review & editing, Supervision, Conceptualization.
